# Investigation, Pollution Mapping and Simulative Leakage Health Risk Assessment for Heavy Metals and Metalloids in Groundwater from a Typical Brownfield, Middle China

**DOI:** 10.3390/ijerph14070768

**Published:** 2017-07-13

**Authors:** Fei Li, Zhenzhen Qiu, Jingdong Zhang, Wenchu Liu, Chaoyang Liu, Guangming Zeng

**Affiliations:** 1 Research Center for Environment and Health, Zhongnan University of Economics and Law, Wuhan 430073, China; zzqiu@zuel.edu.cn (Z.Q.); jdzhang@zuel.edu.cn (J.Z.); lcy@zuel.edu.cn (C.L.); 2 School of Information and Safety Engineering, Zhongnan University of Economics and Law, Wuhan 430073, China; 3 College of Environmental Science and Engineering, Hunan University, Changsha 410082, China; Liuwenchu2015@163.com (W.L.); zgming@hnu.edu.cn (G.Z.)

**Keywords:** brownfield groundwater quality, toxic metals, steady two-dimensional attenuation model, simulative leakage assessment, health risk mapping

## Abstract

Heavy metal and metalloid (Cr, Pb, Cd, Zn, Cu, Ni, As and Hg) concentrations in groundwater from 19 typical sites throughout a typical brownfield were detected. Mean concentrations of toxic metals in groundwater decreased in the order of Cr > Zn > Cu > Cd > Ni > Pb > Hg > As. Concentration of Cr^6+^ in groundwater was detected to further study chromium contamination. Cr^6+^ and Cd in groundwater were recommended as the priority pollutants because they were generally 1399-fold and 12-foldgreater than permissible limits, respectively. Owing to the fact that a waterproof curtain (WPC) in the brownfield is about to pass the warranty period, a steady two-dimensional water quality model and health risk assessment were applied to simulate and evaluate adverse effects of Cr^6 +^ and Cd on the water quality of Xiangjiang River and the drinking-water intake of Wangcheng Waterworks. The results indicated that when groundwater in the brownfield leaked with valid curtain prevention, the water quality in Xiangjiang River and drinking-water intake downstream were temporarily unaffected. However, if there was no curtain prevention, groundwater leakage would have adverse impact on water quality of Xiangjiang River. Under the requirements of Class III surface water quality, the pollution belt for Cr^6+^ was 7500 m and 200 m for Cd. The non-carcinogenic risk of toxic metals in Xiangjiang River exceeded the threshold in a limited area, but did not threaten Wangcheng Waterworks. By contrast, the carcinogenic risk area for adults was at a transverse distance of 200 m and a longitudinal distance of 18,000 m, which was close to the Wangcheng Waterworks (23,000 m). Therefore, it was essential to reconstruct the WPC in the brownfield for preventing pollution diffusion.

## 1. Introduction

Groundwater is one of the most valuable freshwater resources on the earth. It is an essential element of the ecological and geological environment, and has great impacts on biological growth and human life. Some countries have encountered, or are facing groundwater pollution in their developing process. The contamination of groundwater is of serious concern [1]. Toxic metal pollution in groundwater has drawn attention due to strong toxicity, persistence and high bio-accumulation [2]. Moreover, toxic metals have been confirmed to have potential biological risks, ecological risks, and health risks [3,4,5,6]. At present, much research has been carried out to study toxic metal pollution and health risk assessment for groundwater at home and abroad [7,8,9,10].

As a developing country, China is faced with severe groundwater pollution. According to the “Chinese Environmental State Bulletin in 2015”, groundwater in poor quality accounted for approximately 60% of the 5118 monitoring sites in China [11]. In the process of rapid urbanization and industrialization, many factories in the downtown areas and suburbs of cities were moved to industrial districts or were ordered to be shut down by the government. Consequently, a large amount of contaminated sites with unclear contamination levels were left behind, and many contaminated sites were polluted with toxic metals [12,13]. Surface contaminants can enter into groundwater through leaching, leakage, and runoff, and may threaten ecological environment and human health nearby. In recent years, studies characterizing spatial pollution levels and evaluating potential health risk have increased to investigate the pollution characteristics, identify the priority pollutants and areas, and analyze current health risk levels [14,15,16]. However, there are a few studies involving the adverse effects of groundwater leakage in contaminated fields.

The brownfield in this study is a typical toxic metal contaminated site in Middle China. Previous researches have reported that soil in the brownfield is polluted with Cr, As, Pb, Cd and Hg [17,18], so it is of significance to investigate the characteristics of toxic metals in the groundwater of the brownfield. A waterproof curtain (WPC) was built as part of a previous remediation process, but the effective date was limited. Today, the WPC in this brownfield is about to pass the warranty period. The brownfield is located by the side of Xiangjiang River, so groundwater in the brownfield will supply the Xiangjiang River in the dry season if the curtain prevention is ineffective. Furthermore, there are drinking-water intakes of Wangcheng Waterworks 23,000 m downstream. Drinking water in Wangcheng Waterworks will be affected by contamination in Xiangjiang River. Therefore, it is essential to investigate the spatial distribution of toxic metals in the groundwater of the brownfield, and forecast toxic metals concentrations and health risk levels downstream.

The objectives of this study were (i) to investigate the concentrations and spatial distribution of toxic metals in the groundwater from the brownfield, and identify the priority pollutants and areas; (ii) to forecast and analyze toxic metal concentrations in Xiangjiang River under conditions of groundwater leakage with and without WPC, respectively; (iii) to further evaluate health risk of toxic metals on receptors, and judge whether groundwater leakage will threaten drinking water security in the water intake of Wangcheng Waterworks.

## 2. Materials and Methods

### 2.1. Study Area

The brownfield was located in the lower reaches of Xiangjiang River, Middle China (112°56’39”E, 28°15’47”N). The geomorphic unit is the alluvial terrace of the Xiangjiang River, and most of the area is generally level, with the ground elevation between 37.2–44.0 m [18]. The regional geologic structure is mainly made up of sandy conglomerate, aleurolite, sandstone conglomerate, and slate. The geographical location of the studied brownfield belongs to the subtropical monsoon climate zone. The average annual temperature is 17.2 °C and the average annual precipitation is 1362 mm [19]. Paddy soil and red soil are the major soil types in the studied region. The soil textures under different areas of land use from the studied region were generally silt loam, clay, silt clay, silty clay loam, clay loam, sandy loam, and loam [20]. Our previous studies [20,21,22] showed that the soil organic materials (SOM) and pH in the site-specific topsoil were generally 1.8–2.2% (medium to low level in China) and 5.51–6.17 (neutral partial acid soil), respectively.

The predecessor of the brownfield was a chromate factory. The factory covered an area of 150,000 m^2^, with Xiangjiang River in the east, farmlands in the west, a zinc factory in the south, and a chemical factory in the north. It was listed as the second-largest state-owned enterprise in the Chinese chromate industry, with main productions being sodium dichromate and chromic anhydride. The factory supplied raw materials for major industries such as metallurgy, aerospace, and military, and made significant contributions to industrial development. However, the chromate factory discharged a large amount of residue with toxic metals and abandoned them near Xiangjiang River. Cr^6+^ and other toxic metals in the residue could dissolve in surface water, filter into groundwater, leak into Xiangjiang River, and endanger surrounding residents and the environment. Therefore, the factory was forced to shut down in 2003. In 2005, Changsha Chromium Pollution Control Co., Ltd. (Changsha, China) was established to dispose of 420 thousand tons of chromium residue. After detoxification treatment of the chromium residues, a brownfield more than 300 acres was left at the site. Soil and groundwater in the brownfield were seriously polluted by toxic metals, and the pollution has continued to expand over time.

### 2.2. Samples Collection and Detection

**Figure 1:**
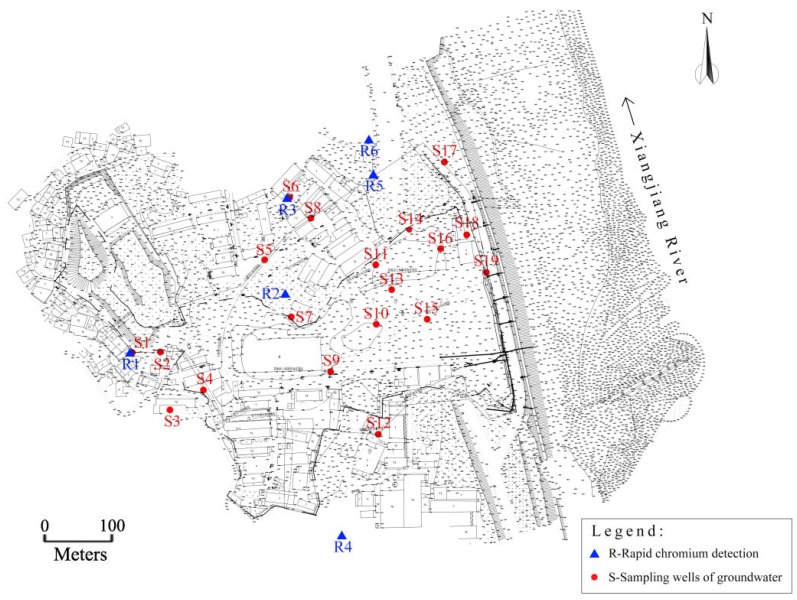
Map of groundwater sampling sites in the brownfield.

A total of 120 groundwater samples were collected throughout the field. Sampling sites were selected based on the distribution of the chromium residue and the location of chromate factory, and verified and confirmed by experts to ensure good experimental design. Designations and locations of sampling sites in this study are presented in Figure 1. Before determination of sampling sites, groundwater samples were collected from sites R1, R2, R3, R4, R5 and R6 for rapid chromium detection, to determine the sampling range. Samples were collected from two depths (5 m and 15 m) in each sampling site to investigate vertical distribution. Afterwards, a total of 120 samples were collected after pumping for 10 min and put into 1 L pre-washed polyethylene containers. The sampling containers were rinsed at least three times with the groundwater before collection. Subsequently, samples for Cr, Pb, Cd, Zn, Cu, Ni and As were acidified to pH 1–2 with HNO_3_ [23], while samples for Hg were acidified with 5 mL HCl [24]. Previous research indicated that soil in the brownfield was severely polluted with Cr [17,18]. Cr^6+^ is the most toxic speciation of chromium, therefore, we conducted a random sampling test for Cr^6+^ in soil from the brownfield during the preliminary field investigation. Results indicated that mean concentration of Cr^6+^ (7138 mg/kg) in the soil significantly exceeded the national secondary standard (30 mg/kg) for commercial and industrial land use [25]. Furthermore, the concentrations of Cr^6+^ in soil extracts from several random sampling sites were much higher than the limit for hazardous wastes, illustrating that the soil in the some parts of the brownfield should be recommended for disposal as hazardous waste [26]. Accordingly, groundwater samples for Cr^6+^ detection were also prepared in order to further study chromium contamination, and samples were alkalized to pH 8 with NaOH [27]. Furthermore, two surface water samples were collected 500 m upstream and 1000 m downstream of the brownfield in Xiangjiang River, and samples were treated similarly to the groundwater samples. In addition, parallel and blank samples were also collected for quality control.

Detection methods of groundwater samples were conducted according to [23,24,27,28]. Cr, Pb, Cd, Zn, Cu and Ni were detected by atomic absorption spectrometry (AAS SP-3520AA, Spectrum, Shanghai, China), Hg was detected by atomic fluorescence spectrometry (AFS-9730, Haiguang, China), while As and Cr^6+^ were detected by ultraviolet-visible spectrophotometry (UV, SP-752, Spectrum, Shanghai, China). Quality assurance and control were carried out with parallel detection experiments, blank tests, and recovery tests.

### 2.3. Steady Two-Dimensional Water Quality Model

A stream water quality model was established based on mass conservation law and the continuity principle of water pollutants [29]. The model reflected movement regularity and the basic characteristics of pollutants in the river. The dimension of the water quality model was studied from a spatial perspective, while the stability of the model was studied from the relationship between time and emission modes. The water quality model could be divided into a non-stable emission model and a stable emission model, according to emission modes (instantaneous emission or continuous emission) [30,31]. A steady two-dimensional attenuation model of non-persistent pollutants was introduced to evaluate the effects of groundwater on Xiangjiang River and Wangcheng Waterworks downstream. The pollutants discharged into Xiangjiang River meet the mass conservation law. If there are gradients of pollutant concentrations in three directions of X, Y and Z, the basic model of environmental quality from three-dimensional space is as follows [32]:

(1) $$\frac{\partial c}{\partial t}{\;+\;\mu }_{x}\frac{\partial c}{\partial x}{\;+\;\mu }_{y}\frac{\partial c}{\partial y}{\;+\;\mu }_{z}\frac{\partial c}{\partial z}{=D}_{x}\frac{\partial^{2}c}{\partial x^{2}}{\;+\;D}_{y}\frac{\partial^{2}c}{\partial y^{2}}{\;+\;D}_{z}\frac{\partial^{2}c}{\partial z^{2}}\;+\;\sum S\mathrm{-kc}\;$$

where c represents the concentrations of pollutants in river (mg/L), μ_x_, μ_y_, μ_z_, represent longitudinal distance, transverse distance and vertical distance (m/s), respectively, t represents time (s); k represents the degradation coefficient (s^−1^); and D_x_, D_y_, D_z_ represent the turbulent diffusion coefficient in the longitudinal direction, transverse direction, and vertical direction, respectively (m^2^/s); ∑S represents the sum of all internal sources and confluences g/(m^3^·s).

Actually, most environmental quality models are complex for obtaining analytical solutions. However, an exploration of the analytical solution is the main method for solving complex models due to the convenient application of the analytical solution. For most models, analytical solution can possibly be obtained under an assumption of a stable and commensurate flow of media. For migration and transformation model of river pollutants, the assumptions are as follows [33]:

(1) Only changes of pollutant concentrations in a longitudinal direction (X) and transverse direction (Y) were took into consideration;
(2) Losses caused by the entrainment effects of sediments were not considered;
(3) Only simplex continuous emission sources were took into account.

Based on the above assumptions, the following derivation model was established. The steady two-dimensional attenuation model is as follows [33,34]:

(2) $$C\left(x,\;y\right)=\exp\left(-k\frac{x}{86,400u}\right)\left\{C_{h}\;+\;\frac{C_{p}Q_{p}}{{H(\pi M}_{y}{\mathrm{xu})}^{\frac{1}{2}}}\left[\exp\left(-\frac{{\mathrm{uy}}^{2}}{{4M}_{y}x}\right)\;+\;\exp\left(-\frac{{u(2B-y)}^{2}}{{4M}_{y}x}\right)\right]\right\}$$

Arbitrary point source emission:

(3) $$C\left(x,\;y\right)=\exp\left(-k\frac{x}{86,400u}\right)\left\{C_{h}\;+\;\frac{C_{p}Q_{p}}{{2H(\pi M}_{y}{\mathrm{xu})}^{\frac{1}{2}}}\left[\exp\left(-\frac{{u(y-y}_{0}{)}^{2}}{{4M}_{y}x}\right)\;+\;2\exp\left(-\frac{{u(2B\;+\;y}_{0}{-y)}^{2}}{{4M}_{y}x}\right)\right]\right\}$$

where C represents the predicted pollutant concentration at any point in the pollution zone (mg/L), C_p_ represents the emission concentration of the pollutant (mg/L), C_h_ represents the pollutant concentration from the upper river (mg/L), Q_p_ represents wastewater flow (m^3^/s). In this study, Q_p_ was 1.26 × 10^−4^ m^3^/s with WPC, and 0.16 m^3^/s without WPC; k represents the attenuation coefficient of pollutant (1/day); M_y_ represents the transverse mixing coefficient of pollutant (m^2^/s); u represents average flow velocity (m/s); H represents the average river depth (m); B represents the river width (m); x represents the distance from the forecast point to the outfall (m); y represents distance from the forecast point to shore (m). The selected values of parameters are shown in Table 1.

### 2.4. Health Risk Assessment

A health risk assessment is identified as the processes used to estimate the probability of events and the probable degree of adverse health effects over a specific period [35]. The risk level of environmental pollutants to a human being depends on the body’s exposure dose to the pollutants and the toxicity of pollutants. There are two main pathways for human being to be exposed to the toxic metals in water: ingestion and dermal absorption, ignoring the exposure via inhalation [5,36,37]. The exposure dose can be calculated by Equations (4) and (5) [38,39].

(4) $${\mathrm{ADD}}_{\mathrm{ing}}=\frac{C_{w}\times \mathrm{IR}\times \mathrm{EF}\times \mathrm{ED}}{\mathrm{BW}\times \mathrm{AT}}$$

where ADD_ing_(μg/(kg·day)) represents the exposure dose through ingestion, in this study, the ingestion mainly refers to the intake through water from Honghu Lake, C_w_ is the mean concentration of toxic metals in water (μg/L), IR is the intake rate of water, including the direct drinking rate and the indirect drinking rate (L/day), in this study 0.81 L/day for a child and 1.79 L/day for an adult [40], EF is the exposure frequency to pollutants (day/year) in this study, 350 day/year [41], ED is the exposure duration (a), meaning the length of time over which contact with the contaminant lasts, in this study, 6 for a child and 30 for an adult [38]; BW represents the body weight (kg), 27.7 kg for child and 58.6 kg for adult [35]; AT is the average time (day), for carcinogenic risk, AT is the average life expectancy of people, 70a for child and adult; for non-carcinogenic risk, AT is equal to ED × 365 [38].

(5) $${\mathrm{ADD}}_{\mathrm{derm}}=\frac{C_{w}{\times \mathrm{SA}\times K}_{p}{\times \mathrm{ET}\times \mathrm{EF}\times \mathrm{ED}\times 10}^{-3}}{\mathrm{BW}\times \mathrm{AT}}$$

where ADD_derm_(μg/(kg·day)) represents the exposure dose through dermal absorption; SA is the exposure area of skin (cm^2^); K_p_ is the dermal permeability coefficient of pollutants in water (cm/h), in this study, 0.001 cm/h for Cd^6+^ and 0.002 cm/h for Cr [5,36]; ET is the exposure time (h/day), in this study, ET was 0.6 h/day [5]. The meanings of C_w_, EF, ED, BW, AT refer to Equation (4).

The health risks caused by environmental pollutants can be divided into carcinogenic risk and non carcinogenic risk according to their properties. Non-carcinogenic risks take the hazard quotient (HQ) as a measure of risk assessment. As shown in Equations (6) and (7) [38], HQ is the ratio of the daily exposure dose to the reference dose. If synergy and antagonism between different pollutants are not considered, integrated non-carcinogenic risk, which is represented by the hazard index (HI), is the sum of HQs caused by various pollutants through different pathways [41]. When HI is greater than 1, it can be considered that there is a certain degree of adverse effects on human health; HI < 1 indicates that there is no harm [38,42].

(6) $${\mathrm{HQ}}_{i}=\frac{{\mathrm{ADD}}_{i}}{{\mathrm{RfD}}_{i}}$$

(7) $$\mathrm{HI}=\overset{n}{\underset{i=1}{\sum }}{\mathrm{HQ}}_{i}$$

where HQ_i_ is the hazard quotient of toxic metals through ingestion or dermal absorption, dimensionless, ADD_i_(μg/(kg·day)) is the daily exposure dose of non-carcinogenic pollutants, RfD (μg/(kg·day)) is the reference dose of pollutants, i is the pathways of exposure, n is the kinds of toxic metals, and HI is the hazard index, which is the sum of HQs of studied toxic metals from all of the applicable pathways; in this study, the pathways included ingestion and dermal absorption of water.

Carcinogenic risk is the product of the daily exposure dose and the cancer slope factor, which are shown as Equations (8) and (9). Under the assumption that there is no antagonism and synergy between pollutants, the integrated carcinogenic risk can also be identified as the sum of carcinogenic risk exposure by various pollutants via different pathways. United States Environmental Protection Agency (USEPA) recommends that the carcinogenic risk level for human being is acceptable within 1 × 10^−4^ [43]. When the carcinogenic risk value is higher than the target risk value (1 × 10^−4^), risk management measures must be taken to protect human health.

(8) $${\mathrm{CR}}_{i}{=\mathrm{ADD}}_{i}{\times \mathrm{CSF}}_{i}$$

(9) $$\mathrm{CR}=\overset{n}{\underset{i=1}{\sum }}{\mathrm{CR}}_{i}$$

where CR_i_ is the carcinogenic risk of toxic metals through ingestion or dermal absorption and is dimensionless, ADD_i_(μg/(kg·day)) is the daily exposure dose of carcinogenic pollutants, and CSF_i_ is the cancer slope factor of carcinogenic pollutants; CR is the sum of CR_i_.

### 2.5. Multivariate and Spatial Analysis Methods

SPSS software (IBM, Armonk, NY, USA) was applied for data entry and data computation. Basic statistical parameters such as range and mean were calculated in order to analyze the characterization of toxic metals. Geographic information system (GIS) data was used to present the pollution distribution of toxic metals in groundwater from the studied brownfield and their simulative leakage health risk levels. Based on the study need, the brownfield status survey and the project cost-benefit consideration, it showed that the total numbers of samples were insufficient to perform such an extensive geostatistical analyses [44,45], but that sample numbers could support reliable spatial analysis, and the spatial analysis methods showed good performance in our previous studies [6,19,45]. Under the consideration of the study requirement and the number of sampling sites, the spatial analysis method (including inverse distance weighted interpolation (IDW), local polynomial interpolation (LP) and radial basis functions interpolation (RBF)) were tested simultaneously to find optimal interpolation method with better parameter uncertainty control. After comparative analysis, the IDW method was finally applied to map the spatial characteristics of regional pollutants. IDW employs a specific number of nearest points that are then weighted according to their distance from the point being interpolated [46,47,48]. In this study, the power of 2 and the number of neighboring samples of 15 were chosen to clearly show both the spatial variation and spatial patterns of the pollutants.

## 3. Results and Discussion

### 3.1. Basic Parameters and Mean Toxic Metal Concentrations in Groundwater from the Brownfield

The pH, total hardness, chemical oxygen demand (COD_Cr_), ammonia nitrogen, sulfide, sulfate, fluoride and nitrate in groundwater from the brownfield are shown as Table 2. The pH ranged from 2.7 to 12, with a mean value of 8.12. There were great differences in pH values among sampling sites, which indicated that the brownfield was significantly disturbed by anthropogenic activities. Groundwater in the central and eastern areas of the brownfield was alkaline, while that in western (S1, S2, S3 and S4) and northern (S5, S6, S8 and S17) areas was acidic. The central and eastern areas were previous stacking points of chromium residue, so groundwater in this area was alkaline owing to influence of chromium residue leachate. Moreover, S1, S2, S3, S4, S5, S6, S8 and S17 were at the edge of the original chromate plant, and these areas were slightly polluted by chromium residue. Groundwater in sites S1–S6, S8 and S17 was acidic, which was consistent with the soil background level (4.32~6.51). The mean content of total hardness was 483.97 mg/L, within the permissible limit of Class III according to the “Chinese Quality Standard for Groundwater” [49]. The maximum value of total hardness was approximately 3.5-fold greater than the limit. Ammonia nitrogen, sulfate, and fluoride in groundwater all exceeded the permissible limit of Class III, and only nitrate was within the limit. The mean content of ammonia nitrogen was approximately 10 times over the permissible limit, while mean content of sulfate was three times the limit and fluoride was five times the limit. Moreover, only 47.4% of the sampling sites were investigated to have sulfide.

Mean toxic metal concentrations in groundwater from the brownfield are shown in Table 3. Mean concentrations of Cr, Pb, Cd, Zn, Cu, Ni, Hg and As in groundwater were 93.06, 0.09, 0.13, 2.14, 1.83, 0.10, 0.001 and 0 mg/L, respectively, for the respective metals listed in descending order: Cr > Zn > Cu > Cd > Ni > Pb > Hg > As. Cr had the highest concentration in groundwater among the studied toxic metals, and it greatly exceeded permit limits of Cr^6+^ according to [49]. Therefore, concentrations of Cr^6+^ were checked to further investigate chromium contamination in groundwater. Compared with the permissible limits of Class III in [49], the mean content of Cr^6+^ was 1399-fold the permissible limit of Class III, and the maximum excessive multiple reached 8799-fold. The mean concentration of Cd was 12-fold the limit of Class III, with a maximum excessive multiple of 47-fold. Furthermore, the mean concentration of Hg was within the limit, while that of Pb, Zn, Cu and Ni were approximately twice the limits. As was not detected in groundwater samples from each sampling site, indicating that groundwater in the brownfield was not contaminated with As. Therefore, Cr^6+^ and Cd were regarded as the priority pollutants in the brownfield. According to the field investigation (Figure S1), contamination in the brownfield has exerted great pressure on environmental quality.

### 3.2. Spatial Distribution of Toxic Metals in Groundwater from the Brownfield

**Figure 2:**
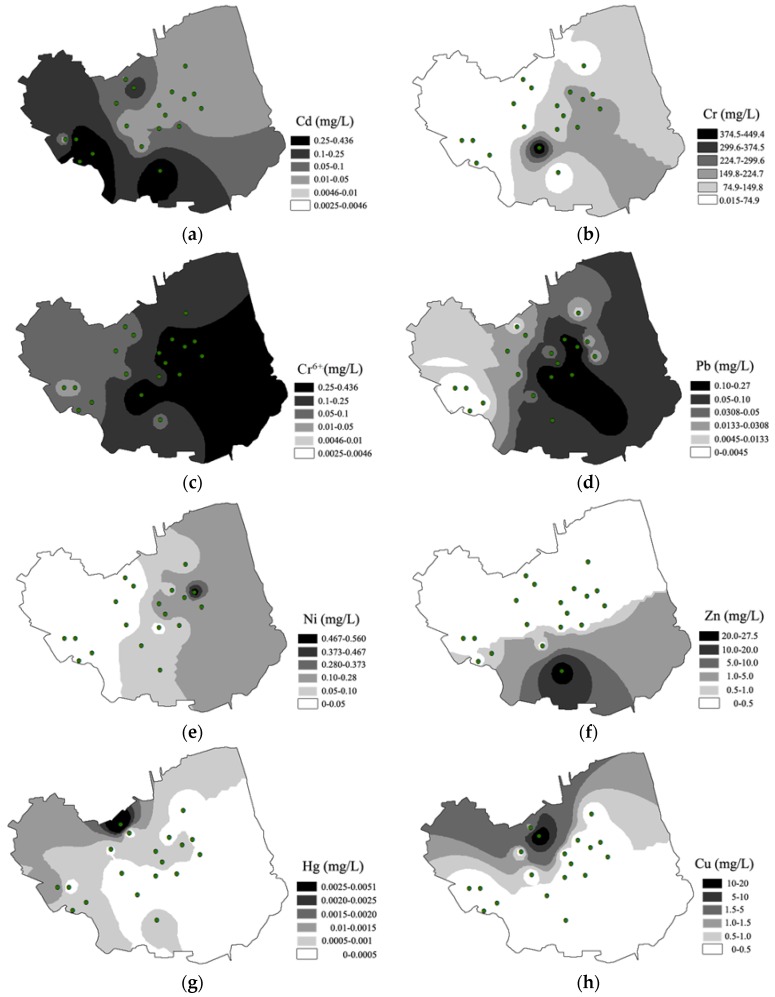
Spatial distribution of Cd (**a**); Cr (**b**); Cr^6+^ (**c**); Pb (**d**); Ni (**e**); Zn (**f**); Hg (**g**) and Cu (**h**) in the groundwater of the brownfield.

Spatial distribution patterns of Cd, Cr, Cr^6+^, Zn, Pb, Ni, Hg and Cu in groundwater from the brownfield are shown in Figure 2a–h using contour maps based on IDW methods. As was not detected in any samples, so distribution mapping of As was not carried out. The maps present the distinct zones of lower or higher concentrations in groundwater. Concentration classification was carried out on maps based on [49].

Based on Figure 2a, the average concentrations of Cd from each sampling site decreased in the order: S12 > S4 > S3 > S2 > S8 > S15 > S10 > S6 > S5 > S11 > S14 > S9 > S17 > S16 > S13 > S18 > S19 > S1 > S7. Moreover, the Cd concentrations of 78.9% of samples exceeded the limits of Class III standards. The southern part and southwest part of the brownfield contained higher Cd concentrations. The sites with higher Cd concentrations were mainly located around the stacking sites for chromium residue, indicating anthropogenic influence.

According to Figure 2b,c, there was a relatively similar spatial distribution between Cr and Cr^6+^. The average contents of Cr in each sampling site decreased in the order: S9 > S15 > S16 > S14 > S19 > S18 > S11 > S13 > S10 > S7 > S8 > S5 > S17 > S6 > S1 > S2 > S3 > S4 > S12, while Cr^6+^ decreased in the order of S9 > S16 > S15 > S19 > S14 > S13 > S18 > S11 > S10 > S7 > S17 > S5 > S1 > S3 > S2 > S4 > S6 > S14 > S8. In combination with Table 3, this indicated that the dominant species of Cr in groundwater was Cr^6+^, and Cr^6+^ has strong toxicity to human health. Cr^6+^ concentrations in 52.6% of samples exceeded the limits of Class III in “Chinese quality standard for groundwater”. East of the brownfield contained an area of higher Cr and Cr^6+^ concentration, while the northwestern area contained lower concentrations. The eastern part of the brownfield had a large stacking point of chromium residue before recommendation of the field, but the western area was the stacking site of treated chromium residue. The results illustrated that removal of Cr from the abandoned chromium residue was quite effective. In addition, the flow direction of groundwater in the brownfield proceeded from west to east along Xiangjiang River, which may increase pollution in the eastern region.

Compared with Cd and Cr, the scales of contamination by other toxic metals were relatively small. The average contents of Pb in each sampling in descending order were: S15 > S10 > S16 > S14 > S13 > S12 > S9 > S7 > S8 > S5 > S11 > S19 > S17 > 0 = S1 = S2 = S3 = S4 = S6 = S18. A total of 31.6% of all sampling sites exceeded corresponding limited concentrations, and another 31.6% were not detected with Pb. Sampling sites with higher Pb concentrations were located in the middle area of the brownfield, which was probably affected by chromium residue. The average contents of each sampling site for Ni were in descending order: S18 > S16 > S9 > S13 > S11 > S12 > S19 > S14 > S15 > S10 > S5 > S7 > S4 > S17 > S3 > S1 > S6 > S2 > S8, with pollution mainly located in the eastern area. By contrast, the Zn contamination of all sampling sites was quite slight, and only concentrations in S12 exceeded the limit. Spatial distributions of Hg and Cu were somewhat similar, with higher concentrations in the north.

**Figure 3:**
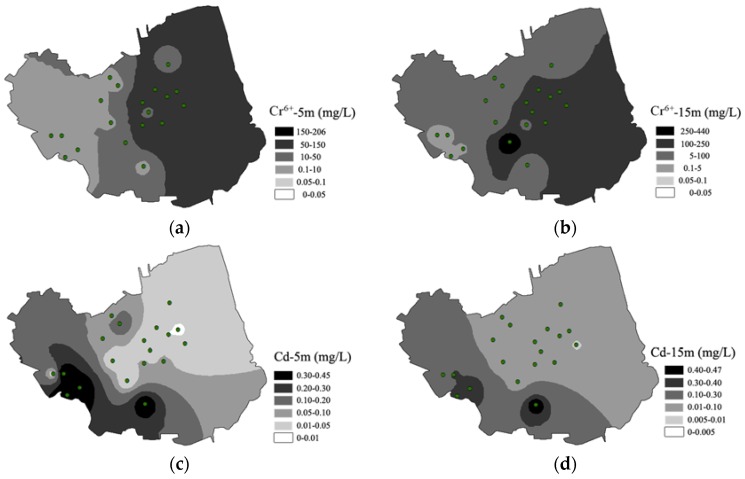
Spatial distribution of Cr^6+^ (**a**,**b**) and Cd (**c**,**d**) in groundwater at depths of 5 m and 15 m.

To better understand the spatial distribution of Cd and Cr^6+^ in groundwater, samples were collected from two depths (5 m and 15 m) in each sampling site (Figure 3). The mean concentrations of Cr^6+^ in groundwater at a depth of 5m decreased in the order: S16 > S15 > S19 > S14 > S18 > S11 > S10 > S9 > S13 > S7 > S5 > S3 > S17 > S4 > S2 > S8 > S6 > S12 > S1, while that at 15 m decreased in the order of: S9 > S13 > S15 > S16 > S14 > S19 > S18 > S11 > S10 > S7 > S17 > S1 > S2 > S5 > S6 > S12 > S3 = S4 > S8. Furthermore, mean concentrations of Cd in groundwater at a depth of 5 m decreased in the order: S3 > S2 > S4 > S12 > S8 > S15 > S6 > S10 > S5 > S14 > S11 > S17 > S19 > S13 > S16 > S7 > S18 > S1 = S9, while that at 15 m decreased in the order: S12 > S4 > S2 > S3 > S8 > S15 > S10 > S9 > S5 > S11 > S6 > S16 > S14 > S17 > S13 > S18 > S1 > S19. The results indicated that spatial distributions of Cd and Cr^6+^ at different depths were relatively similar. Cr^6+^ had spatial distribution patterns of higher concentrations in the east, and lower concentrations in the west throughout, at a depth of 5 m or 15 m. By contrast, sites of higher Cd concentrations were in the western areas of the brownfield, and the average concentrations at the two depths were fairly close. This indicated that groundwater in the ground soil (15 m thick) had strong permeability, and toxic metals could transfer with the flow of groundwater among different depths.

### 3.3. Leakage Effects of Toxic Metals on Water Quality in Xiangjiang River

The original WPC in the brownfield is about to pass the warranty period, which means that diffusion of chromium slag pollution will not be effectively prevented. Without the WPC, the pollutants in groundwater will leak into Xiangjiang River. If Xiangjiang River is polluted, ecological and human health downstream will be threatened, especially in the Wangcheng County. Therefore, a steady two-dimensional water quality model was applied to analyze the adverse effects on downstream under conditions of setting a curtain and not setting a curtain, respectively. The transverse distance was selected as 750 m, and the longitudinal distance was selected as 23,000 m, because the water intake by Wangcheng Waterworks was located 23,000 m downstream.

#### 3.3.1. Effects under the Condition of Setting a Waterproof Curtain

Tables S1 and S2 present the spatial distributions of Cr^6+^ and Cd in Xiangjiang River after groundwater leakage from the brownfield into Xiangjiang River with WPC. As the prediction results shown, after groundwater flowed into Xiangjiang River in dry season, adverse effects of Cr^6+^ and Cd on the water quality of Xiangjiang River can be basically ignored. Concentrations of Cr^6+^ and Cd in the Xiangjing River reached Class I (0.01 mg/L) of the surface water standard [50], except at the leakage point. At the leakage point, the concentration of Cr^6+^ was 0.0477 mg/L, but it was quickly diluted 2000 m downstream. Therefore, Cr^6+^ and Cd had no effect on the water quality of drinking water intake by Wangcheng Waterworks.

Two surface water samples were collected from Xiangjiang River to further support this conclusion. Sampling sites were determined 500 m upstream and 1000 m downstream of the brownfield in Xiangjiang River, respectively. Cr^6+^ was not detected in these two water samples, and Cd was 0.004 mg/L in surface water upstream, but was not detected downstream (Table 4). The results illustrated that water quality in Xiangjiang River was not affected by groundwater leakage with WPC from the brownfield.

#### 3.3.2. Effects under the Condition of Not Setting a Waterproof Curtain

If there was no WPC in the brownfield, concentrations of Cr^6+^ and Cd in Xiangjiang River after groundwater leakage were shown in Tables S3 and S4, respectively. After groundwater in the brownfield flowed into Xiangjiang River, concentrations of toxic metals would decrease due to mixing action and attenuation. When the concentration of Cd in Xiangjiang River reached Class III (0.005 mg/L) [48] of surface water quality requirements, the pollution belt in longitudinal direction would be about 200 m in length. However, the pollution belt of Cr^6+^ in the longitudinal direction was about 7500 m under the same requirement. It was obvious that groundwater leakage had adverse effects on the water quality of Xiangjiang River. When reaching the water intake of Wangcheng Waterworks 23,000 m downstream, concentrations of Cr^6+^ and Cd in surface water of Xiangjiang River would reach Class II and Class I of the surface water standard, respectively. Therefore, groundwater leakage would not have serious impacts on the water quality of Wangcheng Waterworks.

Under the requirement of Class III (0.05 mg/L) [50] water quality in Xiangjiang River, concentrations of Cr^6+^ in the contaminated area decreased in the order: (0, 0) > (2000, 0) > (2000, 50) > (4000, 0) > (4000, 50) > (6000, 0) > (2000, 100) > (4000, 100) > (6000, 50). This phenomenon was quite different from spatial distribution of groundwater leakage with curtain prevention. Longitudinal pollution distance was much longer than transverse pollution distance. The flow direction of groundwater in the brownfield was observed to move from west to east, along the direction of Xiangjiang River. Moreover, the current velocity of the groundwater was 1.4 × 10^−4^ m/s, which was significantly lower than current velocity of Xiangjiang River (0.095 m/s). When there was no WPC, pollutants in groundwater spread into Xiangjiang River in a trumpet shape, which was different from the slow penetration of groundwater with the presence of a curtain.

In summary, the results indicated that after groundwater leakage, concentrations of Cr^6+^ and Cd in surface water from Xiangjiang River had big differences under the conditions of setting a curtain and not setting a curtain. Contaminated groundwater in the brownfield was mainly concentrated in miscellaneous soil, medium-fine gravel, and round gravel. These kinds of soils were characterized by good permeability, and the contaminated groundwater would highly likely leak to the Xiangjiang River. Therefore, it is essential to reconstruct the waterproof curtain in the brownfield.

### 3.4. Leakage Health Risk Assessment of Toxic Metals

Xiangjiang Riveris one of the important drinking water sources in the locality. Water intakes of Wangcheng Waterworks are located 23,000 m downstream of the brownfield. Therefore, it was necessary to evaluate the health risk levels of toxic metals on human health in Wangcheng County. Carcinogenic risks and non-carcinogenic risks of Cr^6+^ and Cd via ingestion and dermal absorption were calculated by Equations (4)–(9).

#### 3.4.1. Non-Carcinogenic Risk Levels

**Figure 4:**
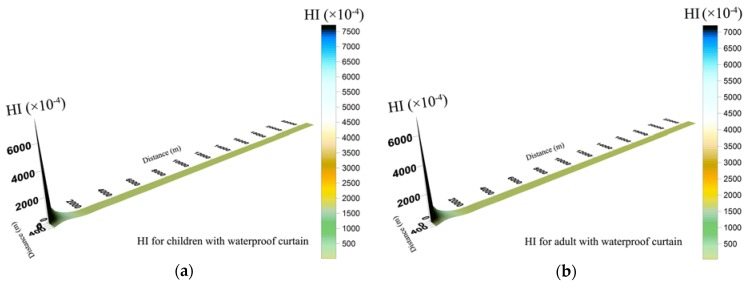
Spatial distribution of hazard index (HI) for children (**a**) and adults (**b**) during groundwater leakage with a waterproof curtain.

Results indicated that integrated non-carcinogenic risk of children was higher than adults. Non-carcinogenic risk of the same toxic metal concentration through drinking water was higher than that through dermal absorption. In other words, the non-carcinogenic risk level exposed by ingestion was higher than by dermal contact, and drinking water intake should be the main pathway of attention. Figure 4 indicated that when groundwater leaked with WPC, the hazard index (HI) of Cr^6+^ and Cd via ingestion and skin were below unity, which means that there were no obvious non-carcinogenic risks to human health. Residents in Wangcheng County are not faced with non-carcinogenic risk caused by contaminated groundwater leakage in brownfield.

**Figure 5:**
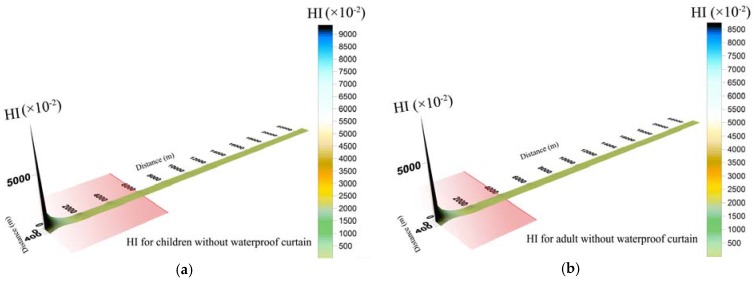
Spatial distribution of hazard index (HI) for children (**a**) and adults (**b**) during groundwater leakage without a waterproof curtain *. * The red region represents that the risk is unacceptable in longitudinal direction, but does not take the transverse range into consideration.

However, when groundwater leaked without curtain prevention (Figure 5), non-carcinogenic risk in some areas of Xiangjiang River exceeded the permissible limit. The hazard index (HI) of children in polluted sites decreased in the order: (0, 0) > (2000, 0) > (2000, 50) > (4000, 0) > (4000, 50) > (2000, 100) > (6000, 50) > 1, while that of adults decreased in the order of (0, 0) > (2000, 0) > (2000, 50) > (4000, 0) > (4000, 50) > 1. Cr^6+^ was recommended as the risk priority pollutant because it generally accounted for 93% of the integrated non-carcinogenic risk. Nevertheless, non-carcinogenic risk exposed by Cr^6+^ and Cd in surface water of Xiangjiang River would not threaten water security in Wangcheng Waterworks.

#### 3.4.2. Carcinogenic Risks Levels

**Figure 6:**
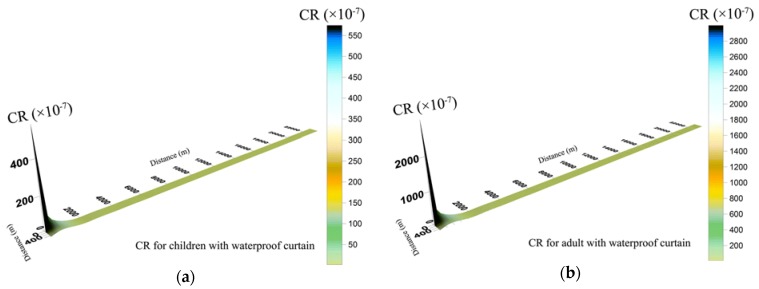
Spatial distribution of carcinogenic risk (CR) for children (**a**) and adults (**b**) during groundwater leakage with a waterproof curtain.

In this study, integrated carcinogenic risk for adults was higher than children. The damage to human health caused by carcinogenic substances is accumulated and hard to be eliminated. When study objects are observed under the same living conditions, the exposure duration of adults is higher than that of children. Therefore, carcinogenic risk for adults was higher than children under identical pollutant concentration and life expectancy. Similarly, the carcinogenic risk of the same toxic metal concentration through drinking water was higher than that through dermal absorption. When groundwater leaked with WPC (Figure 6), the carcinogenic risk of Cr^6+^ and Cd via ingestion and skin were basically within the target risk of 1 × 10^−4^, except at the leakage point. There was no obvious carcinogenic risk to human health in Wangcheng County.

**Figure 7:**
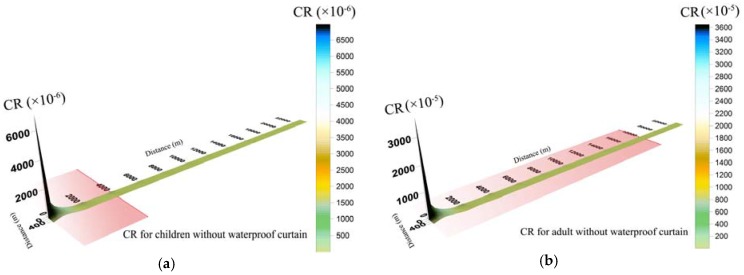
Spatial distribution of carcinogenic risk (CR) for children (**a**) and adults (**b**) during groundwater leakage without a waterproof curtain *. *The red region represents that the risk is unacceptable in longitudinal direction, but does not take the transverse range into consideration.

By contrast, groundwater leakage without WPC prevention caused excess carcinogenic risk in Xiangjiang River (Figure 7). Carcinogenic risk level at the leakage point was much higher than target risk, so groundwater in brownfield should be under relatively frequent monitoring for preventing potential pollution aggravation. Carcinogenic risk area in the Xiangjiang River for adults was larger than that for children. The risk zone for adults was with a transverse distance of 200 m and a longitudinal distance of 18,000 m, which was close to the drinking water intake of Wangcheng Waterworks. The carcinogenic risk in the water intakes of Wangcheng Waterworks was about 7.2 × 10^−5^, which was close to the target risk (1 × 10^−4^). Once the groundwater in brownfield leaked without WPC, toxic metals in groundwater would be likely to threaten the health of adults in Wangcheng County. By contrast, children in Wangcheng County would be much less likely to be exposed tocarcinogenic risk than adults, because carcinogenic risks for children exceeded merely in sites (0, 0), (2000, 0), (2000, 50) and (4000, 0).

### 3.5. Uncertainty Analysis

In this study, a steady two-dimensional water quality model was applied to forecast the adverse effects of Cr^6+^ and Cd on water quality and human health after contaminated groundwater leakage. Owing to the lack of site characteristic parameters and exposure parameters of local residents, there were some uncertainties in the evaluation results. Uncertainties came mainly from the following aspects: (1) Prediction of pollutant concentration. Pollutants would transfer and spread in a transverse dimension, longitudinal dimension and vertical dimension after entering Xiangjiang River. In this paper however, a steady two-dimensional model was selected to simplify the complicated situation, only considering the transverse dimension and the longitudinal dimension. Therefore, there may be some uncertainty in the determination of toxic metal concentrations; (2) Selection of some parameters. Some default parameters were used in the calculation. Default parameters were generally considered to be the most conservative states. The evaluated risk area would probably be larger than the actual risk area; (3) Behavior pattern of research objects. Behavior pattern of the population determines the exposure parameters such as exposure frequency, exposure period and exposure dose. The lack of information on activity patterns of population in this study may have had some uncertainty in the final evaluation of the results.

## 4. Conclusions

Concentrations and spatial distribution of the toxic metals (Cr^6 +^, Cr, Pb, Cd, Zn, Cu, Ni, Hg and As) in groundwater from a typical brownfield were investigated. The mean contents of the studied toxic metals decreased in the order: Cr > Cr^6+^ > Zn > Cu > Cd > Ni > Pb > Hg > As. According to Class III standards, the mean concentration of Hg was within the permissible limit, and Pb, Zn, Cu and Ni were approximately two-fold the limits, with Cd being 12-fold greater than the limit and Cr^6+^ being 1399-fold the limit. Therefore, Cr^6+^ and Cd were regarded as the priority pollutants in the groundwater of the brownfield. The brownfield has been separated by a WPC, but now the WPC is about to pass the warranty period. Once the groundwater leaks without WPC, Xiangjiang River will be polluted by Cr^6+^ and Cd. Predicted pollution belts of Cr^6+^ and Cd were 7500 m and 200 m, respectively, which may adversely affect the aquatic environment in Xiangjiang River. A health risk assessment was conducted to further study adverse effects on human health downstream, especially in Wangcheng County. When the groundwater leaked with WPC, health risk levels for human downstream were within the limits. Conversely, if groundwater leaked without WPC, the health risk on receptors in some areas of Xiangjiang River exceeded the target risk (1 × 10^−4^). Especially, the carcinogenic risk belt for adults was 18,000 m, so people who draw water from these risk areas should pay more attention. Carcinogenic risk in the water intakes of Wangcheng Waterworks was about 7.2 × 10^−5^, which was close to the target risk (1 × 10^−4^). The results proved that WPC around the brownfield was quite effective in reducing pollution and health risk levels. Therefore, it was necessary to reconstruct WPC for cutting off groundwater in the brownfield. The anti-seepage projects can effectively avoid outflow and diffusion of contaminated groundwater, and protect water security for people around the pollution source.

## Figures and Tables

**Table 1:** Selected values of parameters in water quality model.

Parameters	u (m/s)	M_y_ (m^2^/s)	H (m)	B (m)	k (Cr^6+^)·(d^−1^)	k (Cd)·(d^−1^)
Values	0.095	0.154	7.3	794	0.5	0.39

**Table 2:** Physicochemical indexes in groundwater samples (*N* = 38) from the brownfield (mg/L).

Parameters	pH	Total Hardness	COD_Cr_	Ammonia Nitrogen	Sulfide	Sulfate	Fluoride	Nitrate
Mean	8.12	483.97	397.13	2.33	0.052	828.21	5	1.47
Max	12	1510	5480	25	0.17	4180	29.2	8.42
Min	2.7	14	ND ^a^	0.034	ND	ND	ND	ND
Detection limit	2–12	5	5	0.025	0.005	8	0.05	0.02
*N*% ^b^	100	100	81.6	100	47.4	86.8	94.7	92.1
Chinese standard ^c^	6.5–8.5	450		0.2		250	1	20

^a^ ND-not detected; ^b^ *N*%-proportion of observation above detection limit; ^c^ Class III of “Chinese Quality Standard for Groundwater” [49].

**Table 3:** Toxic metal concentrations in groundwater samples (*N* = 38) from the brownfield (mg/L).

Parameters	Cr^6+^	Cr	Pb	Cd	Zn	Cu	Ni	Hg	As
Mean	69.89	93.06	0.09	0.13	2.14	1.83	0.10	0.001	ND
Max	440	841	0.27	0.474	28.8	27.1	0.85	0.00535	ND
Min	ND ^a^	ND	ND	ND	ND	ND	ND	ND	ND
Detection limit	0.004	0.03	0.01	0.0025	0.05	0.0025	0.006	-	0.007
*N*% ^b^	94.7	94.7	57.9	92.1	76.3	57.9	81.6	73.7	0
Chinese standard ^c^	0.05	-	0.05	0.01	1	1	0.05	0.001	0.05

^a^ ND-not detected; ^b^ *N*%-proportion of observation above detection limit; ^c^ Class III of “Chinese Quality Standard for Groundwater” [49].

**Table 4:** Toxic metal concentrations of surface water in Xiangjiang River (mg/L).

Sampling Sites	Cr^6+^	Cr	Pb	Cd	Zn	Cu	Ni
500 m upstream	0.009	ND ^a^	ND	0.004	0.25	ND	ND
1000 m downstream	0.009	ND	ND	ND	ND	ND	ND

^a^ ND: not detected.

## Supplementary Materials

The following are available online at www.mdpi.com/1660-4601/14/7/768/s1, Figure S1: Situation in the studied brownfield, Table S1: Spatial distribution of Cr^6+^ in Xiangjiang River after groundwater flowing into Xiangjiang River with curtain, Table S2: Spatial distribution of Cd in Xiangjiang River after groundwater flowing into Xiangjiang River with curtain, Table S3: Spatial distribution of Cr^6+^ in Xiangjiang River after groundwater flowing into Xiangjiang River without curtain, Table S4: Spatial distribution of Cd in Xiangjiang River after groundwater flowing into Xiangjiang River without curtain.
