# Supplementary material for: Investigation, Pollution Mapping and Simulative Leakage Health Risk Assessment for Heavy Metals and Metalloids in Groundwater from a Typical Brownfield, Middle China
Source: Int J Environ Res Public Health. 2017 Jul 13;14(7):768. doi: 10.3390/ijerph14070768 (PMC5551206; doi:10.3390/ijerph14070768)
Supplement: Supplementary file 1 [file ijerph-14-00768-s001.pdf]

(a) (b) (c)

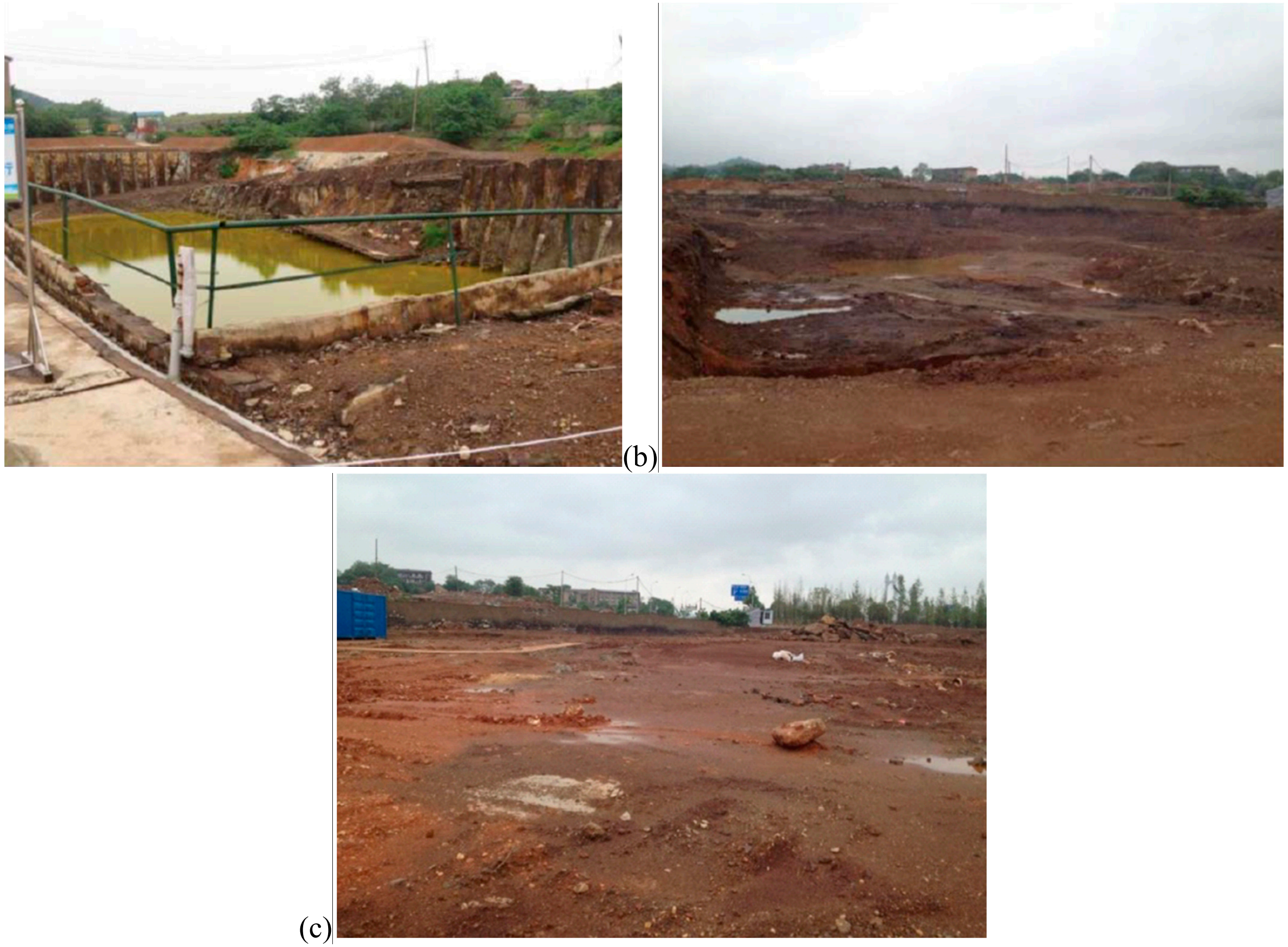

Figure S1. Situation in the studied brownfield [17,18]

Table S1. Spatial distribution of Cr^{6+} in Xiangjiang River after predicted groundwater flow into Xiangjiang River with a curtain

Longitudinal distance (m)[a] / Transverse distance (m)[b]	0	2000	4000	6000	8000	10000	12000	14000	16000	18000	20000	22000	23000
0	0.0477	0.0003	0.0002	0.0002	0.0002	0.0001	0.0001	0.0001	0.0001	0.0001	0.0001	0.0001	0.0001
50	0.0002	0.0003	0.0002	0.0002	0.0002	0.0001	0.0001	0.0001	0.0001	0.0001	0.0001	0.0001	0.0001
100	0.0002	0.0002	0.0002	0.0002	0.0002	0.0001	0.0001	0.0001	0.0001	0.0001	0.0001	0.0001	0.0001
150	0.0002	0.0002	0.0002	0.0002	0.0002	0.0001	0.0001	0.0001	0.0001	0.0001	0.0001	0.0001	0.0001
200	0.0002	0.0002	0.0002	0.0002	0.0001	0.0001	0.0001	0.0001	0.0001	0.0001	0.0001	0.0001	0.0001
250	0.0002	0.0002	0.0002	0.0002	0.0001	0.0001	0.0001	0.0001	0.0001	0.0001	0.0001	0.0001	0.0001
300	0.0002	0.0002	0.0002	0.0002	0.0001	0.0001	0.0001	0.0001	0.0001	0.0001	0.0001	0.0001	0.0001
350	0.0002	0.0002	0.0002	0.0001	0.0001	0.0001	0.0001	0.0001	0.0001	0.0001	0.0001	0.0001	0.0001
400	0.0002	0.0002	0.0002	0.0001	0.0001	0.0001	0.0001	0.0001	0.0001	0.0001	0.0001	0.0001	0.0001
450	0.0002	0.0002	0.0002	0.0001	0.0001	0.0001	0.0001	0.0001	0.0001	0.0001	0.0001	0.0001	0.0001
500	0.0002	0.0002	0.0002	0.0001	0.0001	0.0001	0.0001	0.0001	0.0001	0.0001	0.0001	0.0001	0.0001
550	0.0002	0.0002	0.0002	0.0001	0.0001	0.0001	0.0001	0.0001	0.0001	0.0001	0.0001	0.0001	0.0001
600	0.0002	0.0002	0.0002	0.0001	0.0001	0.0001	0.0001	0.0001	0.0001	0.0001	0.0001	0.0001	0.0001
650	0.0002	0.0002	0.0002	0.0001	0.0001	0.0001	0.0001	0.0001	0.0001	0.0001	0.0001	0.0001	0.0001
700	0.0002	0.0002	0.0002	0.0001	0.0001	0.0001	0.0001	0.0001	0.0001	0.0001	0.0001	0.0001	0.0001
750	0.0002	0.0002	0.0002	0.0001	0.0001	0.0001	0.0001	0.0001	0.0001	0.0001	0.0001	0.0001	0.0001

[a] Longitudinal distance represents the distance from leakage point along the river;

[b] Transverse distance represents the distance from the leakage point to the vertical direction of the river

Table S2. Spatial distribution of Cd in Xiangjiang River after predicted groundwater flow into Xiangjiang River with a curtain

Longitudinal distance (m)[a] / Transverse distance (m)[b]	0	2000	4000	6000	8000	10000	12000	14000	16000	18000	20000	22000	23000
0	0.00011	0.00009	0.00008	0.00008	0.00007	0.00006	0.00006	0.00005	0.00005	0.00004	0.00004	0.00004	0.00003
50	0.00010	0.00009	0.00008	0.00008	0.00007	0.00006	0.00006	0.00005	0.00005	0.00004	0.00004	0.00004	0.00003
100	0.00010	0.00009	0.00008	0.00008	0.00007	0.00006	0.00006	0.00005	0.00005	0.00004	0.00004	0.00004	0.00003
150	0.00010	0.00009	0.00008	0.00008	0.00007	0.00006	0.00006	0.00005	0.00005	0.00004	0.00004	0.00004	0.00003
200	0.00010	0.00009	0.00008	0.00008	0.00007	0.00006	0.00006	0.00005	0.00005	0.00004	0.00004	0.00004	0.00003
250	0.00010	0.00009	0.00008	0.00008	0.00007	0.00006	0.00006	0.00005	0.00005	0.00004	0.00004	0.00004	0.00003
300	0.00010	0.00009	0.00008	0.00008	0.00007	0.00006	0.00006	0.00005	0.00005	0.00004	0.00004	0.00004	0.00003
350	0.00010	0.00009	0.00008	0.00008	0.00007	0.00006	0.00006	0.00005	0.00005	0.00004	0.00004	0.00004	0.00003
400	0.00010	0.00009	0.00008	0.00008	0.00007	0.00006	0.00006	0.00005	0.00005	0.00004	0.00004	0.00004	0.00003
450	0.00010	0.00009	0.00008	0.00008	0.00007	0.00006	0.00006	0.00005	0.00005	0.00004	0.00004	0.00004	0.00003
500	0.00010	0.00009	0.00008	0.00008	0.00007	0.00006	0.00006	0.00005	0.00005	0.00004	0.00004	0.00004	0.00003
550	0.00010	0.00009	0.00008	0.00008	0.00007	0.00006	0.00006	0.00005	0.00005	0.00004	0.00004	0.00004	0.00003
600	0.00010	0.00009	0.00008	0.00008	0.00007	0.00006	0.00006	0.00005	0.00005	0.00004	0.00004	0.00004	0.00003
650	0.00010	0.00009	0.00008	0.00008	0.00007	0.00006	0.00006	0.00005	0.00005	0.00004	0.00004	0.00004	0.00003
700	0.00010	0.00009	0.00008	0.00008	0.00007	0.00006	0.00006	0.00005	0.00005	0.00004	0.00004	0.00004	0.00003
750	0.00010	0.00009	0.00008	0.00008	0.00007	0.00006	0.00006	0.00005	0.00005	0.00004	0.00004	0.00004	0.00003

[a] Longitudinal distance represents the distance from leakage point along the river;

[b] Transverse distance represents the distance from the leakage point to the vertical direction of the river

Table S3. Spatial distribution of Cr^{6+} in Xiangjiang River after predicted groundwater flow into Xiangjiang River without a curtain

Longitudinal distance (m)[a] / Transverse distance (m)[b]	0	2000	4000	6000	8000	10000	12000	14000	16000	18000	20000	22000	23000
0	5.8041	0.1357	0.0854	0.0620	0.0477	0.0379	0.0307	0.0252	0.0209	0.0175	0.0147	0.0125	0.0115
50	0.0020	0.1114	0.0774	0.0581	0.0454	0.0365	0.0297	0.0245	0.0204	0.0172	0.0145	0.0123	0.0113
100	0.0020	0.0628	0.0582	0.0480	0.0394	0.0325	0.0271	0.0226	0.0191	0.0161	0.0137	0.0116	0.0108
150	0.0020	0.0249	0.0364	0.0351	0.0312	0.0270	0.0232	0.0198	0.0170	0.0145	0.0125	0.0107	0.0099
200	0.0020	0.0077	0.0192	0.0229	0.0226	0.0208	0.0187	0.0165	0.0144	0.0126	0.0110	0.0095	0.0089
250	0.0020	0.0028	0.0090	0.0134	0.0150	0.0150	0.0142	0.0130	0.0118	0.0105	0.0093	0.0082	0.0077
300	0.0020	0.0019	0.0041	0.0073	0.0093	0.0102	0.0102	0.0098	0.0092	0.0084	0.0076	0.0069	0.0065
350	0.0020	0.0018	0.0023	0.0039	0.0056	0.0066	0.0071	0.0071	0.0069	0.0065	0.0061	0.0056	0.0053
400	0.0020	0.0018	0.0017	0.0024	0.0033	0.0042	0.0047	0.0050	0.0050	0.0049	0.0047	0.0044	0.0042
450	0.0020	0.0018	0.0016	0.0017	0.0022	0.0027	0.0031	0.0034	0.0036	0.0036	0.0035	0.0034	0.0033
500	0.0020	0.0018	0.0016	0.0015	0.0016	0.0019	0.0021	0.0024	0.0026	0.0026	0.0026	0.0026	0.0025
550	0.0020	0.0018	0.0016	0.0014	0.0014	0.0014	0.0016	0.0017	0.0018	0.0019	0.0020	0.0020	0.0019
600	0.0020	0.0018	0.0016	0.0014	0.0013	0.0012	0.0012	0.0013	0.0014	0.0014	0.0015	0.0015	0.0015
650	0.0020	0.0018	0.0016	0.0014	0.0012	0.0011	0.0011	0.0011	0.0011	0.0011	0.0011	0.0012	0.0012
700	0.0020	0.0018	0.0016	0.0014	0.0012	0.0011	0.0010	0.0010	0.0009	0.0009	0.0009	0.0010	0.0010
750	0.0020	0.0018	0.0016	0.0014	0.0012	0.0011	0.0010	0.0009	0.0009	0.0008	0.0008	0.0008	0.0008

[a] Longitudinal distance represents the distance from leakage point along the river;

[b] Transverse distance represents the distance from the leakage point to the vertical direction of the river

Table S4. Spatial distribution of Cd in Xiangjiang River after predicted groundwater flow into Xiangjiang River without a curtain

Longitudinal distance (m)[a] / Transverse distance (m)[b]	0	2000	4000	6000	8000	10000	12000	14000	16000	18000	20000	22000	23000
0	0.01061	0.00034	0.00024	0.00019	0.00016	0.00014	0.00012	0.00010	0.00009	0.00008	0.00007	0.00006	0.00006
50	0.00010	0.00030	0.00023	0.00019	0.00016	0.00014	0.00012	0.00010	0.00009	0.00008	0.00007	0.00006	0.00006
100	0.00010	0.00020	0.00019	0.00017	0.00015	0.00013	0.00011	0.00010	0.00009	0.00008	0.00007	0.00006	0.00006
150	0.00010	0.00013	0.00015	0.00014	0.00013	0.00012	0.00010	0.00009	0.00008	0.00007	0.00007	0.00006	0.00006
200	0.00010	0.00010	0.00012	0.00012	0.00011	0.00010	0.00009	0.00009	0.00008	0.00007	0.00006	0.00006	0.00005
250	0.00010	0.00009	0.00010	0.00010	0.00010	0.00009	0.00008	0.00008	0.00007	0.00007	0.00006	0.00005	0.00005
300	0.00010	0.00009	0.00009	0.00009	0.00008	0.00008	0.00008	0.00007	0.00007	0.00006	0.00006	0.00005	0.00005
350	0.00010	0.00009	0.00008	0.00008	0.00008	0.00007	0.00007	0.00007	0.00006	0.00006	0.00005	0.00005	0.00005
400	0.00010	0.00009	0.00008	0.00008	0.00007	0.00007	0.00006	0.00006	0.00006	0.00005	0.00005	0.00004	0.00004
450	0.00010	0.00009	0.00008	0.00008	0.00007	0.00007	0.00006	0.00006	0.00005	0.00005	0.00005	0.00004	0.00004
500	0.00010	0.00009	0.00008	0.00008	0.00007	0.00006	0.00006	0.00005	0.00005	0.00005	0.00004	0.00004	0.00004
550	0.00010	0.00009	0.00008	0.00008	0.00007	0.00006	0.00006	0.00005	0.00005	0.00005	0.00004	0.00004	0.00004
600	0.00010	0.00009	0.00008	0.00008	0.00007	0.00006	0.00006	0.00005	0.00005	0.00004	0.00004	0.00004	0.00004
650	0.00010	0.00009	0.00008	0.00008	0.00007	0.00006	0.00006	0.00005	0.00005	0.00004	0.00004	0.00004	0.00004
700	0.00010	0.00009	0.00008	0.00008	0.00007	0.00006	0.00006	0.00005	0.00005	0.00004	0.00004	0.00004	0.00003
750	0.00010	0.00009	0.00008	0.00008	0.00007	0.00006	0.00006	0.00005	0.00005	0.00004	0.00004	0.00004	0.00003

[a] Longitudinal distance represents the distance from leakage point along the river;

[b] Transverse distance represents the distance from the leakage point to the vertical direction of the river
